# Self reported skin morbidity and ethnicity: a population-based study in a Western community

**DOI:** 10.1186/1471-5945-7-4

**Published:** 2007-06-29

**Authors:** Florence Dalgard, Jan Øivind Holm, Åke Svensson, Bernadette Kumar, Johanne Sundby

**Affiliations:** 1 Institute of General Practice and Community Medicine, University of Oslo, Norway; 2 Department of Dermatology, Ullevål University Hospital, Oslo, Norway; 3 Department of Dermatology, University Hospital, Malmø, Sweden

## Abstract

**Background:**

Recent studies have shown ethnic differences concerning cardio-vascular disease, diabetes and mental health. Little is known about ethnic differences in skin morbidity. The purpose of this study was to describe possible ethnic differences in self-reported skin morbidity in a Western urban community.

**Methods:**

The design was cross sectional. 40 888 adults in Oslo, Norway, received a postal questionnaire providing information on socio-demographic factors and self-reported health, including items on skin complaints.

**Results:**

18770 individuals answered the questionnaire. In the sample 84% were from Norway. The largest immigrant group was from Western countries (5%) and the Indian Subcontinent (3%). Itch was the most prevalent reported skin symptom (7%), and was significantly more reported by men from East Asia (18%) and Middle East/North Africa (13%). The same observations were seen for reported dry and sore skin. Hair loss was a dominating complaint for men from the Indian Subcontinent and the Middle East/North Africa (23% and 25%) and for women from the same ethnic groups. Women from Sub-Saharan Africa reported significantly more pimples than in the other groups (17%).

**Conclusion:**

The study showed that there were significant differences in self-reported skin complaints among ethnic groups. Issues concerning the cultural value of some skin symptoms should be examined further.

## Background

Human migration is an increasing phenomenon, people move to cities in the West, both from rural areas but also from other parts of the globe contributing to a multiethnic context[1]. In Norway migration from developing countries is recent.

The term "Ethnicity" has been revisited and a debate exists in the medical literature [2-4]. Research in this field has been challenging because of a lack of uniformly-accepted standards in defining race and ethnicity[5]. In dermatology the classification of populations according to their external characteristics such as skin color has been the custom[6] but this classification has not taken into consideration aspects as history of migration, birthplace, language and religion[3,7,8]. Although there is little evidence for ethnic differences regarding the structure and function of the skin[9], the spectrum of skin diseases among ethnic groups seen in dermatology clinics seems to be different[10]. Furthermore, the self-description of the perceived effects of skin disease touches on cultural context because skin diseases are visible and often stigmatizing, and the weight of stigma might vary in different ethnic groups[11,12].

There are few epidemiological studies related to skin disease and ethnicity among adults, most relate to child populations [13-16]. Ethnic differences in skin morbidity among adults in Western communities have been reported in a publication from patient records from dermatological departments in four European cities [17]. It showed that eczema and fungal infections were more prevalent among Asians, and another study from Leicester report that pigment disorders, pruritus and atopic dermatitis were more prevalent among Asians than non-Asians[18]. A study from south-east London showed that acne was most frequently seen among black patients (African, Afro-Caribbean and mixed) [10]. Although skin diseases are common in the community, most of our knowledge on skin diseases in the general population is based on Western populations and is biased through strong selection into hospital-based samples and specific diagnoses[19]. Recently, a questionnaire for population survey on self-reported skin disease was validated making it possible to examine skin morbidity in the community[20]. It has been shown that self-reported skin morbidity is associated with poor general health, socio economic factors and psycho-social factors in the general population [21-23].

It has been shown that immigrant mortality is higher in England and Wales[24] and that the health status of Ethnic minorities is worse, concerning self-assessed general health, psycho-social health, cardio-vascular disease, obesity, diabetes and respiratory disease[25]. The aim of this present study was to answer the following questions: are there ethnic differences in self-reported skin morbidity among adults in an urban Norwegian community?

## Methods

### Participants

The study was cross sectional. In 2000–2001 the Oslo Health Study was conducted under the joint collaboration of the Norwegian Institute of Public Health, the University of Oslo and the Municipality of Oslo. The study population included all individuals in Oslo County born in 1970, 1960, 1955, 1940/41 and 1924/25. A total of 40 888 individuals were invited to participate and received a postal questionnaire. Details are described in a previous publication[21]. The questionnaires provided information on socio-demographic factors, self-reported health, various aspects of health behavior, and psycho-social factors. Of the persons invited the total number participating in this study was 18747 (42.4% men and 49.9% women, in total 45.9%). In addition the missing percentage for each question varied between 22.2% and 24.8% for skin complaints.

### Variables

Socio-demographic variables included household income, social class, and education, all self- reported variables. Social class was based on employment and categorized in five, the highest class represented by higher grade professionals, followed by lower grade professionals, routine non-manuals (administration, commerce, sales services), skilled manual workers, semi and unskilled workers (including housewives, students, unemployed, retired, disabilities benefits).

Norwegian population registers identify all residents with a unique identification code and includes country of birth in addition to other demographic data. This was used as the basis of the invitation file. The ethnicity variable was based on the country of birth and was further classified into eight groups taking into account migration history, religion, language and diet habits. The one hundred fifteen different nationalities represented in the sample were classified as follows:

1. Norway as the reference population

2. Western countries (mostly from Scandinavian countries, UK, Germany, USA)

3. Eastern Europe (mostly from Bosnia-Herzegovina, Hungary)

4. Indian Subcontinent (mostly from Pakistan, Sri Lanka)

5. East Asia (mostly from Vietnam)

6. Sub-Saharan Africa (mostly from Somalia)

7. Middle East/North Africa (mostly from Turkey, Iran)

8. Central/South America (mostly from Chile)

Socio economic status was measured by self-reported household income, with low income (< 300000 Nkr), middle income (300–500000 Nkr), and higher income (> 500000 Nkr).

The skin questionnaire was developed and validated in Norwegian and presented in a previous publication[20].

### Statistic analysis

SPSS statistical package version 11-0 was used. The data were analyzed with frequencies, cross tables with Chi Square test.

### Ethics

The study protocol was reviewed by the Regional Committee for Medical Research Ethics and approved by the Norwegian Data Inspectorate. The study has been conducted in full accordance with the World Medical Association's Declaration of Helsinki.

## Results

### Population characteristics

The socio-demographic characteristics of the Oslo population with age, sex, marital status and ethnic groups are presented in table 1. The age distribution of the sample was uniform, with a slight overweight of individuals between 40 and 45 years. There were more females than males and half of all lived alone. Among the minorities, individuals from other Western countries predominated followed by individuals from the Indian Subcontinent. The smallest ethnic groups were from Sub Saharan Africa and Central/South America.

**Table 1:** Population characteristics of the sample of Oslo N = 18770

**Variables**	**No**	**%**
Age(years)		
30	4106	21.9
40/45	6594	35.2
59/60	4469	23.8
75/76	3578	19.1
Sex		
Male	8392	44.8
Female	10355	55.2
Marital status		
Living alone	9276	49.8
Not living alone	9363	50.2
Ethnicity*		
Norway	15797	84.3
Western countries	1025	5.5
Eastern Europe	259	1.4
Indian Subcontinent	605	3.2
East Asia	336	1.8
Sub-Saharan Africa	209	1.1
Middle East/North Africa	406	2.2
Central and South America	110	0.6

* The dominating countries in each category were as follows: Western countries: Denmark, Sweden, UK, Germany, USA; Eastern Europe: Bosnia-Herzegovina, Hungary; Indian Subcontinent: Pakistan and Sri Lanka; East Asia: Vietnam; Sub-Saharan Africa: Somalia; Middle East/North Africa: Turkey, Iran; Central and South America: Chile.

As more than 80% of all ethnic groups lived in their home country before age 16, this population sample of Oslo residents consists mainly of first generation immigrants (data not shown).

### Socio-economic distribution among ethnic groups

Table 2 shows that there were no differences among Norwegians and individuals from Western countries as far as social class and income was concerned. Individuals from Western countries and Eastern Europe were higher educated than Norwegians and all other ethnic groups. In all other ethnic groups social class III dominated.

**Table 2:** Socio-economic distribution among ethnic groups* in the sample of Oslo N = 18770

	**Ethnic groups**							
	**Norway**	**Western countries**	**Eastern Europe**	**Indian Subcontinent**	**East Asia**	**Sub-Saharan Africa**	**Middle East/North Africa**	**Central and South America**

	**N = 15797 (%)**	**N = 1025 (%)**	**N = 259 (%)**	**N = 605 (%)**	**N = 342 (%)**	**N = 195 (%)**	**N = 406 (%)**	**N = 110 (%)**

Social class								
I (Highest)	23	24	18	5	9	9	11	17
II	13	14	10	2	7	6	4	11
III	53	52	47	43	49	44	40	40
IV	5	5	6	7	8	8	7	3
V	6	5	18	43	26	32	38	29
Education Men^§^								
Lower secondary	35	32	31	56	50	24	33	40
Higher secondary	32	32	39	32	30	46	31	28
University	32	37	39	11	20	29	35	32
Education Women								
Lower secondary	41	21	18	52	28	41	20	24
Higher secondary	33	21	42	33	35	29	53	39
University	26	38	40	15	36	29	27	36
Household income								
< 300000 Nkr**	30	30	48	60	42	62	62	38
300–500000 Nkr	31	32	32	31	36	26	30	35
> 500000 Nkr	38	38	20	9	22	12	8	27

* The dominating countries in each category were as follows: Western countries: Denmark, Sweden, UK, Germany, USA; Eastern Europe: Bosnia-Herzegovina, Hungary; Indian Subcontinent: Pakistan and Sri Lanka; East Asia: Vietnam; Sub-Saharan Africa: Somalia; Middle East/North Africa: Turkey, Iran; Central and South America: Chile.

** < annual income, 1 Euro = 7,7 Nkr

^§ ^Men N = 8392, Women N = 10355, see table one population characteristics.

Immigrants from the Indian Subcontinent were poorest educated, but the education level was high among immigrants from East Asia, Sub Saharan Africa, Middle East/North Africa and Central/South America.

Men were less educated than women in all ethnic groups except among Norwegians. More women than men have university education except women from the Middle East/North Africa and from Norway.

Norwegians and the other Western individuals have the same pattern of household income and they have a significantly higher income compared with the other ethnic groups. The lowest reported household income was among immigrants from Indian Subcontinent, Sub- Saharan Africa, and Middle East/North Africa. In all eight categories the percentage of those having middle income was appreciatively the same.

### Prevalence of self-reported skin complaints within ethnic groups for each gender separately

There was significantly more reporting of itch among men from East Asia (18% versus 7% among Norwegians) and men from the Middle East and North Africa (13%), the same observation for the report of dry and sore skin. There were no ethnic differences in reporting scaly skin and pimples among men. Men from Middle East/North Africa reported significantly more sweat (14% vs. 4%). Hair loss is the dominating complaint for men from Indian Subcontinent 23% vs. 3%) and Middle East/North Africa (25%).

There was no significant ethnic difference among women for reporting itch, scaly skin or hand rash. Women from Sub-Saharan Africa report significantly more pimples than Norwegians (17% vs. 4%). Reported sweat is significantly increased among women from Eastern Europe and Middle East/North Africa (16% and 16% versus 4%). Women from The Indian Subcontinent, Sub-Saharan Africa and Middle East/North Africa report significantly more hair loss compared to Norwegian women (respectively 31%, 20%and 29% vs. 3%).

There is a tendency that women reported more skin complaints than men among all ethnic groups with the exception of East Asia.

## Discussion

This population based survey provides a contribution to the field of health and ethnicity, reflecting the spectrum of reported skin morbidity among immigrants in a Western community.

The main findings are that there are ethnic and gender differences in the reporting of skin complaints. Itch is a common symptom to many chronic skin diseases like eczemas and urticaria. It is a challenging symptom because of its subjectivity, and little is known about the prevalence of this symptom in the general population. This study is the first describing the prevalence among ethnic groups of reported itch. Some reports stated differences in skin permeability and barrier function in Africans and Asians[26], but no epidemiological studies were found in the literature.

Hand rash, as hand eczema, is commonly known as a skin disease predominating among women[27], but in this study East Asian men complain significantly more about itch, dry, sore skin and hand rash than men from other ethnic groups. Most probably this is due to their occupation. Unfortunately it was not possible to get information about the occupation and exposure in the survey. It might be possible that those men work longer; with wet work and that they have several jobs. Another explanation could be that East Asian men are more predisposed to eczema than others, the study from immigrants in Leicester points out an increased prevalence of atopic eczema and pruritus among Asians[17]. But in that study Asians are not specifically defined and refer probably both to the Indian Subcontinent and South East Asia. It is therefore difficult to compare the results with our findings.

Women from Sub Saharan Africa, but also East Asia and Middle East/North Africa report significantly more pimples than Norwegian women; on the other hand there are no significant ethnic differences among men. Our study points out gender differences in self- reported pimples. The findings confirms the results of a study describing skin diseases in South-East London showing that acne is most prevalent among black adults in an unstratified analysis[10]. Likely the answers to the question asking for pimples include hyperpigmentation problems often following treated or untreated acne in darker skin[28]. The high prevalence rates of pimples among some women from ethnic minorities could indicate an increased importance in some cultures that women should have a problem free skin, and a concern for stigmatization because of skin problems[11,29].

The high prevalence of reported hair loss among men and women from the Indian Subcontinent, East Asia and Middle East/North Africa is a striking finding in this investigation. In a study among immigrants in European cities it is stated that among individuals from Cape Verde, Turkey, Morocco and Indonesia hair loss (alopecia) was a dominating diagnosis[17]. There are several hypotheses for these differences. An explanation could be genetic differences, i.e. individuals from the Indian Subcontinent and the Middle East/North Africa could have more dense hair and probably might be predisposed to loose hair under new climatic circumstances, or new diet habits. Another hypothesis could be that the instrument measuring mental health could be less valid among some ethnic groups and that the reported hair loss could be associated with stress and be the expression of somatization. Nevertheless, the high report of hair loss indicates a concern that is different in Northern parts of the world compared to Asia, Africa or the Middle East/North Africa. Abundant hair has a tremendous status both for men and women in many parts of the world reflecting beauty and health.

There are some limitations to the study. Firstly the used questionnaire on self-reported skin morbidity was elaborated for use in the general population and not for clinical purposes. Secondly it was not constructed especially for ethnic minorities, so that particular problems related to dark skin like hypo-or hyper pigmentation were not included in the questionnaire. Furthermore the instrument was validated in a Norwegian sample but not among non-Western individuals which adds to the difficulty to interpret the responses from the latter group[20].

The low response rate and the additional proportions of missing from the questionnaire is a major concern. Such a high loss clearly affects the representativeness of the sample and the validity of the final estimates. However a non-responder study was conducted based on a linkage between socio-demographic data from public registers in Statistics Norway and data from this study. The study concluded that "self-selection according to socio-demographic variables had little impact on prevalence estimates but an overestimation of the odds ratios of chronic disease among persons born in non-Western countries would be expected", we should therefore be prudent in the conclusions of our findings[30]

## Conclusion

We have shown that there are significant differences among ethnic minorities in a Western population in self-reported skin morbidity. This study is a contribution to research on Health and Ethnicity. Issues concerning the cultural value of some skin symptoms should be examined further.

## Competing interests

The author(s) declare that they have no competing interests.

## Authors' contributions

FD and JS designed the study. FD and ÅS carried out the analysis and drafted the paper. FD, JØH and BK participated actively in the discussion. All authors commented on draft versions and approved the final manuscript.

**Table 3:** Prevalence of reported skin complaints within ethnic groups in the sample of Oslo for each gender separately, N = 18770

**Men**	**Ethnic groups**								SignificanceP**
	**Norway**	**Western countries**	**Eastern Europe**	**Indian Subcontinent**	**East Asia**	**Sub-Saharan Africa**	**Middle East/North Africa**	**Central and South America**	

	**N = 7020 %**	**N = 410 %**	**N = 108 %**	**N = 312 %**	**N = 138 %**	**N = 112 %**	**N = 245 %**	**N = 47 %**	

**Skin complaints %**									
Itch	7	8	8	11	18	3	13	9	< 0.001
Dry/sore skin	6	6	6	9	18	9	15	5	< 0.001
Scaly skin	5	5	1	4	10	5	7	5	Ns
Hand rash	2	2	4	1	7	3	9	5	< 0.001
Pimples	2	2	1	3	4	3	3	5	Ns
Face rash	1	1	1	2	7	3	5	5	< 0.001
Warts	1	1	1	5	1	0	3	5	< 0.001
Sweat	4	3	10	11	10	2	14	10	< 0.001
Hair loss	3	3	7	23	11	7	25	0	< 0.001
Other skin complaints	2	2	10	6	10	5	12	9	< 0.001

**Women**	**Ethnic groups**								**SignificanceP**

	**Norway**	**Western countries**	**Eastern Europe**	**Indian Subcontinent**	**East Asia**	**Sub-Saharan Africa**	**Middle East/North Africa**	**Central and South America**	

	**N = 8777 %**	**N = 615 %**	**N = 151 %**	**N = 293 %**	**N = 198 %**	**N = 97 %**	**N = 161 %**	**N = 63 %**	

**Skin complaints %**									
Itch	9	10	11	15	12	14	16	11	Ns
Dry/sore skin	6	8	10	11	9	13	10	7	< 0.001
Scaly skin	4	4	6	5	2	3	3	2	Ns
Hand rash	4	4	5	5	6	6	6	2	Ns
Pimples	4	5	6	7	8	17	9	5	< 0.001
Face rash	2	2	7	3	3	6	4	5	< 0.001
Warts	1	1	2	2	1	9	6	0	< 0.001
Sweat	4	4	16	7	2	11	16	4	< 0.001
Hair loss	3	3	14	31	14	20	29	7	< 0.001
Other skin complaints	3	3	9	3	4	6	12	0	< 0.001

** Level of significance between the ethnic groups.

## Pre-publication history

The pre-publication history for this paper can be accessed here:


